# The General Management of Fractures

**Published:** 1875-10

**Authors:** A. B. Crosby


The General Management of Fractures—*By A. B. Crosby, M.D.*—Your patient having reached his home, the question arises, when is the permanent dressing to be applied? or in other words, *when is the broken bone to be set?* I am surprised that some surgeons have been found who advocate leaving a fracture practically without dressing for some days, upon the theory that the material necessary for the repair of a broken bone is not thrown out until seven to ten days following the occurrence of the injury, and therefore that it is a matter of indifference whether the dressing is applied before that time or not. Such a theory is exceedingly pernicious. The sooner a fracture is dressed the better it is for the patient; for if you leave it subject to irregular muscular contraction, there will be more or less laceration of the soft parts by the ends of the fragments, irritation will be produced, and undue inflammation will be much more likely to be present. It seems to me that the question is settled beyond discussion, and that the surgeon who neglects to reduce a fracture as soon as possible after it has been received, and secure it in position by proper dressings, is certainly guilty of a misdemeanor, if not of actual crime. * * *

Other things being equal, a person can lie longer, without suffering serious inconvenience, upon a hard than upon a soft bed. If the patient is suffering from a fracture of the spine, or has suffered from any injury which is accompanied with great loss of power, and will necessitate the parts below to be subjected to persistent pressure, we are very apt to have the ordinary results which follow long-continued pressure and loss of vitality, namely, bedsores. It is not safe, therefore, to attempt the treatment of a patient who has received a fracture or other serious injury of the spine upon an ordinary bed. For this purpose the best bed that can be employed is the air or water bed. This consists of an india-rubber bag or flattened sac of the requisite dimensions, which can be filled with air or water, which ever you may prefer to employ. In either case the sac should be only moderately distended, that is, it should be left a little yielding, so that, when covered with a blanket, it will yield just enough to pressure to fit the back, or whatever part of the body may come in contact with it, perfectly and equally throughout; for perfectly equable pressure never resulted in a bedsore. It is only persistent unequal pressure upon prominent points which is likely to give rise to bedsore, and if the same persons, in most cases, who have bedsores, had been as persistently in one position upon an air or water bed, such sores would not have occurred.

The management of the dejections from a person who is confined to the bed with a fracture sometimes becomes a serious matter. It is for this reason that many surgeons, when treating a fracture of the lower extremity, have one thick mattress, in the central portion of which is a round or square opening, and in the floor of the bedstead a trap-door that can be opened or closed at pleasure from the under side. The cavity in the mattress is filled with a cushion sufficiently thick so that no depression is left in the surface when it is in place, and when the patient wishes to have a movement from the bowels it falls out as the trap-door is unfastened. With such a convenience he may have a dejection in comparative peace. It is usually necessary to have a urinal independent of the vessel which is used beneath this opening in the bed, and the greatest care must be exercised in keeping the mattress around the opening, and also the patient's clothes, perfectly dry and clean.

It does not always happen, however, that you can have such a mattress and the arrangement I have just alluded to, and the next best arrangement that can be employed is the ordinary dust-pan. Cover such a pan with a folded sheet or towel, having previously pushed a towel before it for the purpose of protecting the dressings upon the thigh, and then raising up the sound leg the thin edge of the dust-pan can be easily carried well under the body of the patient and receive the dejection. If the discharge is so copious as to be likely to soil the clothing, the patient should arrest it, the pan be removed, a fresh sheet applied, and then replaced. The dust-pan can be placed beneath the patient much easier than the bed-pan, as, if the latter is used, you will be much more likely to disturb the fracture, while the former answers a most admirable purpose. * * *

The question of bandages is one upon which, first and last, there has been a great diversity of opinion. The two forms of bandage in common use are the ordinary roller bandage and the bandage of Scultetus. Now the question arises, when to use a bandage, and where to use a bandage; and these are very important.

I think it may be laid down as a law that the roller bandage never should be carried around the limb over the seat of fracture, unless you are applying some permanent dressing.

The reason for this rule is that if you are using a movable dressing, one which is to be removed to enable you to get at the fracture, you cannot do it without the greatest inconvenience if you employ the roller bandage.

The roller bandage, however, should be applied usually from the tips of the extremities up over the limb for a certain distance. * * With fracture of the humerus, the roller bandage may be carried over the forearm to the elbow, for the purpose of preventing the occurrence of swelling below the seat of fracture after the permanent dressings have been applied. With reference to the lower extremity, the same principle holds good; and with fracture of the thigh, you will be obliged to bandage the foot, ankle, and leg to the knee, and then leave the seat of fracture uncovered by this form of bandage.

In fractures of the lower extremity, however, there is no objection to applying over the seat of fracture the bandage of Scultetus, which is the most beautiful bandage ever devised, and the one that affords the most equable compression. This bandage can be applied and laid over the limb without disturbing the line of fracture, and that cannot be done with the common roller bandage.

The bandage of Scultetus is made in the following manner:

First, tear a strip of old cloth about two and one-half inches wide and as long as the part you desire to cover. Next tear several strips, about the same width and long enough to go once and a half around the thigh, or whatever limb you may be treating. Now, having laid the strip in one direction, take one of the strips which are to go around the limb, and place it across the first strip at right angle, the middle of the latter strip corresponding to the middle line of the former. A second transverse strip is then laid upon the first, overlapping it by one-half its width, and so you will go on until the strip, representing the length of the part to be covered, is itself covered by these transverse pieces. These pieces should then be fastened by two rows of stitches at the point where they intersect each other. The bandage is then complete and is applied to the posterior surface of the limb with the single strip running lengthwise. Then beginning at the lower strip one end is passed around the limb, while the other is made to overlap it, and the second strip follows this, and so on until the last one is turned, which is to be fastened with a pin, and the entire bandage is secured. When you wish to examine the limb, all that is necessary to do is to remove the outside splints, unfasten the upper strip, and turning back one end after another you soon reach the bare surface. Now by such palpation as is necessary you can determine whether or not the fragments are being retained in proper relation with each other.

There is one caution which should always be borne in mind by the surgeon when dressing a fracture, and that is, *all* dressings, in order to be borne and to be useful, must compress the limb in all places equably. If you are to apply a bandage over an extremity up to near the seat of fracture, it must be applied in such a manner as will give a perfectly equable and firm pressure. If there is any difference to be made, it should be the firmest at the tip of the extremity and less firm as you go up the limb ; but as a rule, the more equable the pressure the better it will be borne, and the better will be the result.—*Medical Record.*

